# A Prediction Model Based on Noninvasive Indicators to Predict the 8-Year Incidence of Type 2 Diabetes in Patients with Nonalcoholic Fatty Liver Disease: A Population-Based Retrospective Cohort Study

**DOI:** 10.1155/2021/5527460

**Published:** 2021-05-14

**Authors:** Xintian Cai, Qing Zhu, Yuanyuan Cao, Shasha Liu, Mengru Wang, Ting Wu, Jing Hong, Ayguzal Ahmat, Xiayire Aierken, Nanfang Li

**Affiliations:** Hypertension Center of People's Hospital of Xinjiang Uygur Autonomous Region, Xinjiang Hypertension Institute, National Health Committee Key Laboratory of Hypertension Clinical Research, Urumqi, China

## Abstract

**Background:**

The prevention of type 2 diabetes (T2D) and its associated complications has become a major priority of global public health. In addition, there is growing evidence that nonalcoholic fatty liver disease (NAFLD) is associated with an increased risk of diabetes. Therefore, the purpose of this study was to develop and validate a nomogram based on independent predictors to better assess the 8-year risk of T2D in Japanese patients with NAFLD.

**Methods:**

This is a historical cohort study from a collection of databases that included 2741 Japanese participants with NAFLD without T2D at baseline. All participants were randomized to a training cohort (*n* = 2058) and a validation cohort (*n* = 683). The data of the training cohort were analyzed using the least absolute shrinkage and selection operator method to screen the suitable and effective risk factors for Japanese patients with NAFLD. A cox regression analysis was applied to build a nomogram incorporating the selected features. The C-index, receiver operating characteristic curve (ROC), calibration plot, decision curve analysis, and Kaplan-Meier analysis were used to validate the discrimination, calibration, and clinical usefulness of the model. The results were reevaluated by internal validation in the validation cohort.

**Results:**

We developed a simple nomogram that predicts the risk of T2D for Japanese patients with NAFLD by using the parameters of smoking status, waist circumference, hemoglobin A1c, and fasting blood glucose. For the prediction model, the C-index of training cohort and validation cohort was 0.839 (95% confidence interval (CI), 0.804-0.874) and 0.822 (95% CI, 0.777-0.868), respectively. The pooled area under the ROC of 8-year T2D risk in the training cohort and validation cohort was 0.811 and 0.805, respectively. The calibration curve indicated a good agreement between the probability predicted by the nomogram and the actual probability. The decision curve analysis demonstrated that the nomogram was clinically useful.

**Conclusions:**

We developed and validated a nomogram for the 8-year risk of incident T2D among Japanese patients with NAFLD. Our nomogram can effectively predict the 8-year incidence of T2D in Japanese patients with NAFLD and helps to identify people at high risk of T2D early, thus contributing to effective prevention programs for T2D.

## 1. Introduction

The global prevalence of adult diabetes has increased rapidly in recent decades and has become a major public health problem [1]. Type 2 diabetes (T2D), the most common form of diabetes, is a chronic disease characterized by elevated blood glucose levels due to insufficient insulin production and insulin resistance [2]. In addition, there is growing evidence that nonalcoholic fatty liver disease (NAFLD) is associated with an increased risk of diabetes, independent of traditional diabetes risk factors [3, 4]. As a debilitating chronic epidemic, a core component of T2D prevention strategies is to identify individuals at high risk of T2D [5]. Studies have shown that early identification of people at high risk for T2D and timely lifestyle changes or pharmacological interventions can delay the process of *β*-cell failure and the development of T2D [6, 7]. Therefore, it is important to study the risk factors for the development of T2D in Japanese patients with NAFLD and to find an easy, reliable, and accurate screening tool for the identification of T2D high-risk groups in NAFLD. This will contribute to the effective implementation of T2D prevention programs in Japanese adults with NAFLD.

Risk prediction models have great potential in the decision-making process for the management of subhealthy populations and patients [8, 9]. Risk prediction models can help guide screening and interventions to predict the onset of diseases. At present, a variety of risk prediction models have been constructed to identify individuals at high risk of T2D, such as Leicester Risk Assessment [10], the Cambridge Risk Score [11], QDiabetes® Calculator [12], and the FINDRISC [13], but they all have a number of limitations. First, most do not take into account lifestyle changes such as physical activity, smoking, and alcohol consumption behaviors. Others are based on invasive and cost-effective data, or small-scale and inappropriate cohort selection. Others are based on short-term follow-up or lack of transparent reporting on the steps that produced the pattern. Most importantly, these diabetes prediction models are based on the general population and limited research focused on individuals at low risk.

The purpose of this paper is to develop a T2D risk prediction model for Japanese patients with NAFLD based on data from the NAGALA cohort study to better screen and assess the 8-year risk of developing T2D in high-risk nondiabetic patients.

## 2. Materials and Methods

### 2.1. Data Source

We have downloaded the raw data uploaded by Okamura et al. from the “DATADRYAD” database (http://www.datadryad.org) for free. Since Okamura et al. [14] have granted the data Dryad website ownership of the raw data, we were able to use them for secondary data analysis according to different scientific hypotheses (Dryad data package: 10.5061/dryad.8q0p192).

### 2.2. Data Description

The raw data variables in the database file included baseline information, incident T2D, and follow-up time. The following variables were extracted: incident T2D, follow-up duration, gender, age, waist circumference (WC), BMI (body mass index), fatty liver, alcohol consumption, smoking status, exercise habits, aspartate transaminase (AST), alanine aminotransferase (ALT), high-density lipoprotein cholesterol (HDL-C), gamma-glutamyl transferase (GGT), triglycerides (TG), total cholesterol (TC), fasting plasma glucose (FPG), hemoglobin A1c (HbA1c), systolic blood pressure (SBP), and diastolic blood pressure (DBP).

### 2.3. Study Design and Participants

In this study, Okamura et al. [14] used the NAGALA (NAfld in the Gifu Area, Longitudinal Analysis) database to investigate the effect of obesity phenotype on the risk of developing T2D. Since most of the participants require repeated examinations, the researchers conducted a follow-up study of incident T2D diagnosed by blood tests and fatty liver diagnosed by abdominal ultrasound. This study was a secondary analysis of the open data from the NAGALA study. The inclusion criteria for the NAGALA study were described in detail in the original article [15]. Briefly, a total of 15744 participants were selected according to the following exclusion criteria: (1) a lack of important data; (2) the participants had hepatitis B or C virus or fatty liver disease; (3) alcohol intake exceeding 60 g per day for men or 40 g per day for women; (4) the participants took medication at baseline; (5) fasting plasma glucose ≥ 6.1 mmol/L.

### 2.4. Data Collection and Measurements

In the initial study, a standardized self-administered questionnaire was used to survey all participants about their medical history and lifestyle factors, including physical activity, alcohol consumption, and smoking habits. Researchers assessed alcohol consumption by asking participants about the type and amount of alcohol consumed per week in the previous month and then estimated the average weekly alcohol consumption. Participants were divided into the following four groups: no or light drinking, <40 g/week; light drinking, 40-140 g/week; moderate drinking, 140-280 g/week; or heavy drinking, >280 g/week. The researchers also divided the participants into three groups based on their smoking status: never smokers, past smokers, or current smokers. Nonsmokers were defined as participants who never smoked, past smokers were defined as participants who smoked in the past but quit before the baseline examination, and current smokers were defined as participants who smoked at the time of the baseline examination. Researchers also investigated participants' recreational and physical activities. The researchers defined regular exercisers as participants who participated in any type of exercise at least once a week.

### 2.5. Definitions

T2D was defined as HbA1c ≥ 48 mmol/mol, FPG ≥ 126 mg/dL, and/or self-reported diabetes during follow-up. NAFLD was defined as having fatty liver demonstrated by abdominal ultrasound.

### 2.6. Ethical Approval

As this study is the second analysis of existing anonymous data, informed consent of participants is not required. Published paper details the ethical permission [14].

### 2.7. Statistical Analyses

The study is consistent with the transparent report of the multivariate predictive model of individual prognosis or diagnosis (TRIPOD): the TRIPOD statement [16].

Statistical analyses were performed using R software (version 3.6.3; https://www.R-project.org). First, 2741 Japanese patients with NAFLD were randomly divided into a training cohort of 2058 and a validation cohort of 683 for external validation using the R-caret package, consistent with a theoretical ratio of 3 : 1. Data were expressed as mean ± standard deviation (normal distribution) or median (quartile) (skewed distribution) for continuous variables, and categorical variables were evaluated by calculating frequencies or percentages. Two-sample *t*-tests were used to analyze differences between the training and validation cohorts for normally distributed continuous variables, the Wilcoxon rank-sum test for nonnormally distributed continuous variables, and the chi-square test for categorical variables. And then, the data of the training cohort were analyzed using the least absolute shrinkage and selection operator (LASSO) method to screen the suitable and effective risk factors for Japanese patients with NAFLD. LASSO regression is a method to simplify high-dimensional data. Features with nonzero coefficients can be selected in the LASSO regression model. Next, indicators selected in the LASSO regression model were included in the univariate and multivariate cox regression analysis of risk factors related to T2D, and the hazard ratio (HR) and 95% confidence interval (CI) were calculated. The results of the univariate and multivariate cox regression analyzes were visualized using forest plots. Finally, results of the multivariate cox regression analysis were used to construct a nomogram prediction model. In addition, a variety of validation methods were used to estimate the accuracy of the risk prediction model by using data from the training and validation cohorts, respectively. C-index and receiver operating characteristic (ROC) curve were used to quantify the discrimination performance of nomogram. We plotted and calculated calibration curves using the rms software package, which was used to evaluate the calibration of the T2D risk nomogram and accompanied by a Hosmer-Lemeshow test. Decision curve analysis was performed to determine the clinical application of the T2D risk prediction model: the proportion of true positive results minus the proportion of false positive results, and then, the relative risks of false positive and false negative results were weighted to obtain the net benefits of decision-making. Bootstraps for 1000 resample were performed on the ROC curve, C-index, calibration curve, and decision curve analysis to reduce overfitting deviation. Survival analysis was also performed using the Kaplan-Meier analysis between low-risk and high-risk groups according to the cut-off value of 50%, and the log-rank test was performed to compare survival variance in different groups. All statistical tests were two-sided, and *P* values of <0.05 were considered significant.

## 3. Results

### 3.1. Baseline Characteristics of the Study Cohort

**Figure 1:**
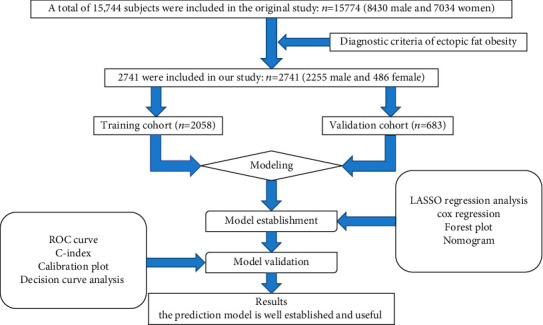
Flow diagram of study design.

A total of 2741 participants were included in this study, of which 2058 were in the training cohort and 683 were in the validation cohort. A flow diagram of studying design is depicted in Figure 1. The overall incidence of T2D was 8.14% (223/2518). In the training and validation cohorts, the incidence of T2D was 157 (7.63%) and 66 (9.66%), respectively. The median follow-up time for the training cohort was 1865 days (quartile: 779-3445), and the median follow-up time for the validation cohort was 2073 days (quartile: 1054-3474). In addition, there were no significant differences observed between the two cohorts. Baseline characteristics of training and validation cohorts are summarized in Table 1.

### 3.2. Characteristics of Selection by LASSO Regression Analysis

**Figure 2:**
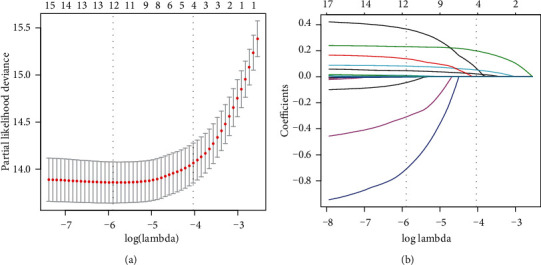
Demographic and clinical feature selection using the LASSO regression model. (a) Tenfold cross-validated error (first vertical line equals the minimum error, whereas the second vertical line shows the cross-validated error within 1 standard error of the minimum). (b) LASSO coefficient profiles of all the clinical features. A coefficient profile plot was produced against the log(lambda) sequence. Each of the different colored curves in the figure represents the trajectory of each independent variable coefficient. The vertical coordinate is the value of the coefficient, the lower horizontal coordinate is log(lambda), and the upper horizontal coordinate is the number of nonzero coefficients in the model. LASSO: least absolute shrinkage and selection operator; SE: standard error.

Seventeen potential risk factors were selected from demography and clinical characteristics and analyzed by LASSO regression (Figures 2(a) and 2(b)). Nonzero characteristic variables were selected based on the statistical approach of the LASSO regression model. Therefore, the number of potential variables was reduced from seventeen to four, including smoking status, WC, FPG, and HbA1c.  Table 2 shows the specific coefficients corresponding to the variables of lambda.1se.

### 3.3. Univariate and Multivariate cox Regression Analysis in the Training Cohort

**Figure 3:**
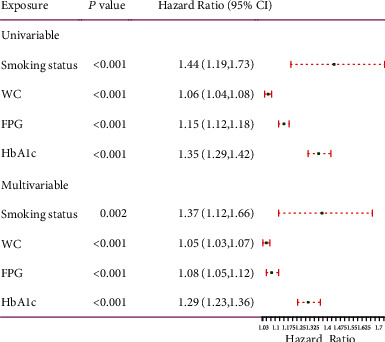
Forest plot of univariable and multivariable cox regression analysis for risk factors associated with T2D. HR: hazard ratio; CI: confidence interval.

Univariate and multivariate cox regression analyses (Figure 3(a) of univariate analysis and Figure 3(b) of multivariate analysis) were performed on 2058 Japanese patients with NAFLD in the training cohort. The results showed that smoking status, WC, FPG, and HbA1c were considered to be independent predictors of T2D (*P* < 0.05).

### 3.4. Development of the Individualized Prediction Model

**Figure 4:**
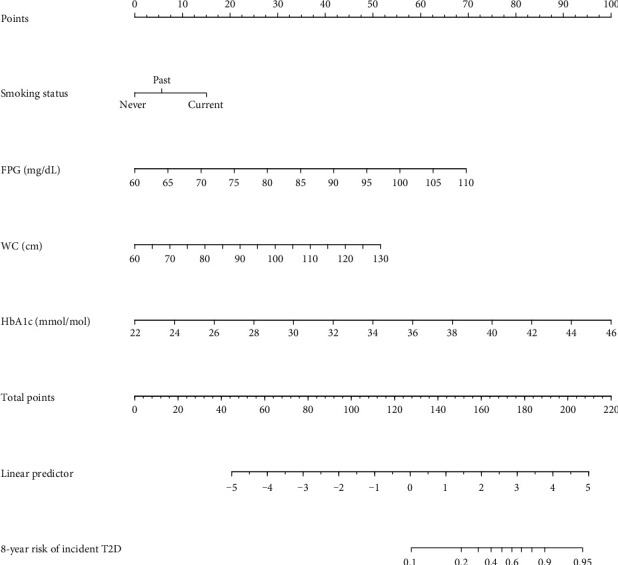
Nomogram for predicting the 8-year risk of T2D in adults with NAFLD. To use the nomogram, an individual patient's value is located on each variable axis, and a line is drawn upward to determine the number of points received for each variable value. The scores for all variables are then added to obtain the total score, and a vertical line is drawn from the total-points row to estimate the 8-year risk of T2D in adults with NAFLD at the lower line of the nomogram.

We have combined the above four independent predictors into a predictive model and displayed it in the form of a nomogram. As shown in Figure 4, the nomogram is a quantitative and convenient tool. To obtain a personalized 8-year risk of T2D in Japanese patients with NAFLD, a vertical line was drawn from the values on the point scale to assess these points, which were then summed to obtain values for each variable. The sum includes the total score and matches the risk on the bottom axis.

### 3.5. Performance of the Nomogram

**Figure 5:**
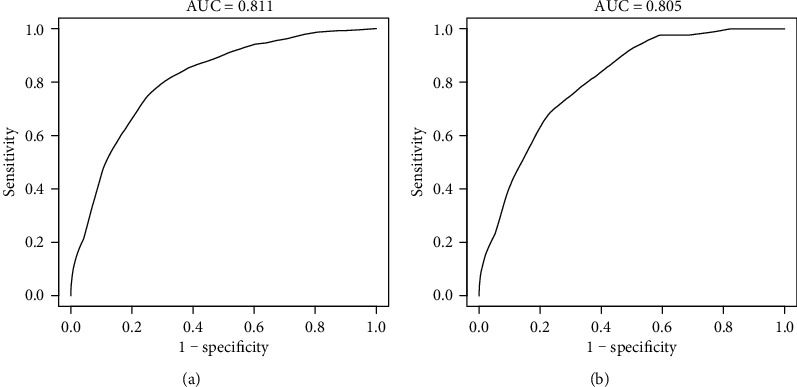
ROC curve of the nomogram in the training and validation cohort. (a) ROC curve of the nomogram in the training cohort. (b) ROC curve of the nomogram in the validation cohort. ROC: receiver operating characteristic; AUC: area under the curve (bootstrap resampling times = 1000).

**Figure 6:**
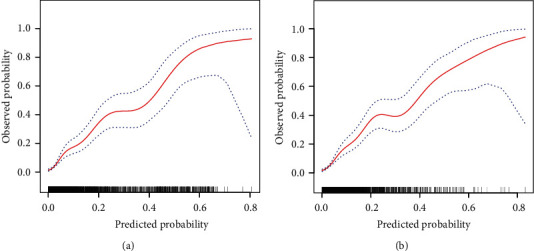
Calibration curves for the training and validation cohort models. (a) Calibration curve of the nomogram in the training cohort. (b) Calibration curve of the nomogram in the validation cohort. The red curve is a calibration curve corresponding to the actual situation. The blue curve represents the 95% CI range of the calibration curve (bootstrap resampling times = 1000).

**Figure 7:**
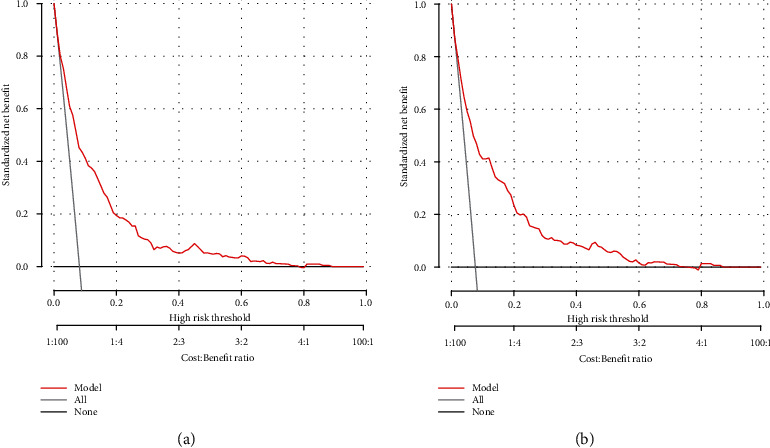
Decision curve analysis of the nomogram in the training (a) and validation cohorts (b). The *y*-axis stands the net benefit. The *x*-axis indicates the threshold probability. The red line represents the nomogram. The black line displays the net benefit of the strategy of treating no patients. The gray line displays the net benefit of the strategy of treating all patients (bootstrap resampling times = 1000).

**Figure 8:**
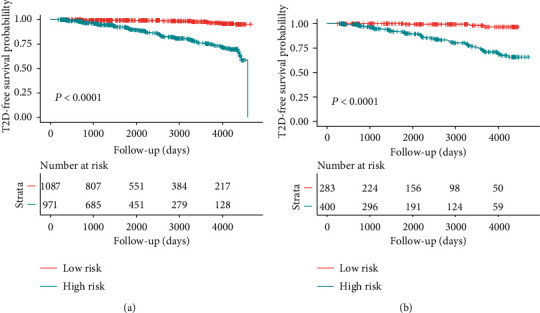
Kaplan-Meier curves of risk group stratification based on the predictor from nomogram prediction. Kaplan-Meier curves show the T2D-free survival probability of high risk (blue) and low risk (red) groups between the training cohort (a) and the validation cohort (b). Risk tables are also added.

The ROC curve and C-index were used to evaluate the discriminatory ability of the prediction model. For the prediction model, the pooled area under the ROC of the nomogram was 0.811 with a sensitivity and specificity of 77.49% and 72.36%, respectively, in the training cohort (Figure 5(a)). It was 0.805 in the validation cohort, with sensitivities and specificities of 69.88% and 75.59%, respectively (Figure 5(b)), which indicates a moderately good performance. The C-index of training cohort and validation cohort was 0.839 (95% CI, 0.804-0.874) and 0.822 (95% CI, 0.777-0.868), respectively. As shown in Table 3, the nomogram showed a good prediction model. The prediction model was calibrated using the calibration curve and the Hosmer-Lemeshow test. From the calibration curves, the prediction model showed a good fit in both the training and validation cohorts (Figures 6(a) and 6(b)). As shown by the Hosmer-Lemeshow test, there was good agreement between the predicted and actual probabilities in both the training and validation cohorts. The decision curve analysis of the training cohort (Figure 7(a)) and the validation cohort (Figure 7(b)) indicated that the application of the prediction model in Japanese patients with NAFLD to predict the risk of T2D incidence is more effective than the intervention-for-all-patients scheme. Each patient was divided into a high-risk or low-risk group according to the cut-off value of 50% predicted by nomogram. Kaplan-Meier survival analysis yielded a significant difference in T2D-free survival probability between the training cohort (Figure 8(a)) and the validation cohort (Figure 8(b)). This stratification could effectively discriminate the T2D prevalence outcomes of the two risk groups in the training and validation cohort.

## 4. Discussion

With rapid economic development, human lifestyles have changed dramatically worldwide. The prevalence and incidence of T2D are rapidly increasing worldwide [17]. T2D is associated with an increased risk of cardiovascular disease and premature death. It is the main cause of end-stage renal disease, blindness, and nontraumatic amputations resulting from microvascular complications, thus imposing a significant economic burden on society [18, 19]. The risk of T2D is strongly associated with lifestyle, nutritional status, and environmental factors [20]. Several large intervention studies have shown that lifestyle changes or pharmacological interventions targeting people at risk of T2D can effectively prevent or delay the onset of T2D and reduce the risk of death from T2D and its complications [21, 22]. The key to successful intervention is the early identification of people at high risk of T2D [18]. In recent years, numerous T2D risk prediction models have been developed and tested, such as the Australian Type 2 Diabetes Risk Assessment Tool [23], the Cambridge Risk Score [11], the Finnish Risk Score [13], the Framingham Diabetes Risk Score [24], and among others [25–27]. However, there is a lack of a risk prediction model based on cohort study data for the Japanese population, especially for patients with NAFLD. Most of the available T2D risk prediction models are only applicable to the target population, and direct application of these models constructed mainly from populations of European origin may underestimate the risk of developing T2D in the Japanese population. Based on data from the NAGALA cohort study, we aimed to develop a T2D risk prediction model for Japanese patients with NAFLD to identify individuals with high risk of T2D.

In this retrospective cohort study, we developed and validated a nomogram model using cost-effective and easily available parameters to predict the 8-year risk of T2D in Japanese patients with NAFLD and to help clinicians identify high-risk populations for T2D. In the training and validation cohorts, our nomogram has excellent prediction performance and also has excellent consistency on the calibration curve. The decision curve analysis illustrates the clinical application value of nomogram. To the best of our knowledge, this study is the first nomogram to use continuous values instead of segmented values to estimate the risk of type 2 diabetes in Japanese patients with NAFLD. In addition, the nomogram will be of great practical value due to its easily available parameters.

Our prediction model includes four parameters: smoking status, WC, FPG, and HbA1c. These variables identified as risk factors for T2D were consistent with previous studies [28, 29]. In our nomogram, current or past smokers have a higher risk of developing T2D than never-smokers. Numerous epidemiological studies have confirmed that smoking is associated not only with the occurrence of T2D but also with the increased risk of T2D hospitalization and mortality, and the risk increases in a dose-dependent manner with the increase of daily smoking [30–32]. According to the 2014 US secretary of health report, compared with nonsmokers, smoking increases the risk of T2D of active smokers by 30-40%, which indicates that smoking cessation should be emphasized as a basic public health strategy to combat the global diabetes epidemic [33]. The World Health Organization also recognizes that smoking is a preventable risk factor for T2D and agrees to avoid smoking/quit smoking as part of its lifestyle recommendations [30], although there is a lack of a complete understanding of the potential pathways of tobacco abuse, especially the mechanism of pancreatic beta cells. However, data from numerous clinical studies suggest that smoking and nicotine have effects on body composition, pancreatic beta cell function, and peripheral insulin sensitivity [20, 29, 34, 35].

Obesity has become a major global epidemic affecting more than 300 million people, and it is the most important risk factor leading to the onset of T2D [36, 37]. BMI has been used as a surrogate marker of obesity and as one of the predictor variables in most diabetes risk models [38–41]. However, BMI does not reflect central obesity. Compared with BMI, WC has a better predictive value for incident T2D, which is consistent with the results of this study [42–44]. WC is a simple anthropometric parameter of abdominal obesity; it is an indicator of central obesity, whereas BMI is an indicator of general obesity [45, 46]. Central obesity is a recognized risk factor for the metabolic syndrome and is strongly associated with the secretion of adipocytokines and inflammatory cytokines, all of which are strongly associated with an increased risk of developing T2D [47–49]. Elevated WC always leads to an accumulation of abdominal fat and a subsequent increase in free fatty acid levels [50]. Excess of circulating free fatty acids leads to insulin resistance by inhibiting insulin signalling and directly accelerating the rate of hepatic gluconeogenesis, coupled with desensitization of the hepatic regulatory loop involving fatty acids by hypothalamic sensing [51–53]. Therefore, waist circumference was used as the basis for the construction of the predictive model in this study.

The FPG level can reflect the secretion level and function of basal insulin [54]. Many epidemiological studies have shown that baseline FPG levels are highly predictive of T2D, and the elevated baseline FPG levels are closely related to the increased risk of developing T2D [55–58]. HbA1c is a comprehensive measurement method of circulating blood glucose level, which reflects the average blood glucose level over the previous 2-3 months and is used as the gold standard for long-term follow-up of blood glucose control [59, 60]. Compared with the oral glucose tolerance test (OGTT) and 2hPG, the measurement of HbA1c is faster and more convenient and can be measured at any time, regardless of the length of fasting or the composition of the previous meal [61, 62]. Epidemiological studies show that elevated HbA1c in nondiabetic adults is associated with T2D incidence rate, incidence rate of cardiovascular disease, and mortality [63–65]. A recent recommendation by the American Diabetes Association Committee on the Diagnosis and Classification of Diabetes Mellitus advocates the use of HbA1c as a practical and effective test method in the diagnosis of prediabetes to identify high-risk groups, and it may be cost-effective to carry out intensive lifestyle interventions to prevent T2D. The decision of the American Diabetes Association Committee is mainly based on the established association between HbA1c and microvascular diseases [59].

Although our nomogram performed well, several limitations warrant mention. First, we relied on FPG and HbA1c, rather than OGTT, to define incident T2D. However, OGTT is not feasible to carry out this test in a large sample. HbA1c does not need fasting, reflecting the long-term blood glycemic status. In addition, the International Expert Committee also recommended the use of HbA1c to diagnose diabetes [66]. Second, our validation cohort was derived from the same population as the training cohort, which may indicate that the findings were overly optimistic. Future research could verify the performance of this prediction model with other databases. Third, this large-scale cohort study was conducted in Japan. Therefore, whether the results of this study can be generalized to other ethnic groups and some specific groups, such as pregnant women and children, requires further validation by external cohorts. Fourth, the current assessment results might not be satisfactory in practice, and some novel biochemical markers or indicators, especially of genetic factors, could improve the performance of the prediction model in the future. Finally, this report is a second analysis based on the existing database. Although many confounding factors have been adjusted, these potential predictors were not included in our prediction model because data on socioeconomic status, lifestyle (except for smoking, drinking, and exercise habits), disease history (such as cardiovascular disease and chronic kidney disease), family health history (such as diabetes), and specific timing of diabetes diagnosis (especially for self-reported diabetes) were not collected in the database.

## 5. Conclusion

In summary, we developed and validated a nomogram for the 8-year risk of incident in T2D among Japanese patients with NAFLD, including smoking status, WC, FPG, and HbA1c. Our prediction model can effectively predict the 8-year incidence of T2D in Japanese patients with NAFLD and helps to identify people at high risk of T2D early, thus contributing to effective prevention programs for T2D.

## Figures and Tables

**Table 1:** Demographic and clinical characteristics of the study population in the training and validation cohorts.

Variables	Total	Training cohort	Validation cohort	*P* value
No. of participants	2741	2058	683	
Age (years)	44.80 ± 8.29	44.98 ± 8.34	44.27 ± 8.14	0.054
BMI (kg/m^2^)	25.48 ± 3.10	25.48 ± 3.08	25.50 ± 3.18	0.898
WC (cm)	86.10 ± 7.75	86.09 ± 7.64	86.13 ± 8.07	0.706
ALT (IU/L)	27.00 (20.00-39.00)	27.00 (20.00-39.00)	26.00 (20.00-38.00)	0.843
AST (IU/L)	22.54 ± 9.94	21.00 (17.00-26.00)	20.00 (16.00-25.50)	0.805
GGT (IU/L)	23.00 (17.00-35.00)	23.00 (17.00-35.00)	23.00 (16.00-34.00)	0.139
HDL-C (mmol/L)	1.19 ± 0.30	1.19 ± 0.29	1.19 ± 0.31	0.564
TC (mmol/L)	5.44 ± 0.86	5.45 ± 0.85	5.43 ± 0.92	0.559
TG (mmol/L)	1.25 (0.88-1.82)	1.25 (0.88-1.82)	1.25 (0.85-1.82)	0.526
HbA1c (mmol/mol)	34.34 ± 3.65	34.36 ± 3.66	34.29 ± 3.63	0.648
FPG (mg/dL)	97.32 ± 6.56	97.27 ± 6.60	97.48 ± 6.44	0.464
SBP (mmHg)	123.77 ± 14.79	124.01 ± 14.85	123.07 ± 14.59	0.152
DBP (mmHg)	78.13 ± 10.18	78.28 ± 10.19	77.66 ± 10.14	0.169
Gender (*n* (%))				0.635
Female	486 (17.73%)	369 (17.93%)	117 (17.13%)	
Male	2255 (82.27%)	1689 (82.07%)	566 (82.87%)	
Habit of exercise (*n* (%))				0.313
No	2340 (85.37%)	1765 (85.76%)	575 (84.19%)	
Yes	401 (14.63%)	293 (14.24%)	108 (15.81%)	
Alcohol consumption (*n* (%))				<0.001
None	2088 (76.18%)	1574 (76.48%)	514 (75.26%)	
Light	286 (10.43%)	232 (11.27%)	54 (7.91%)	
Moderate	250 (9.12%)	179 (8.70%)	71 (10.40%)	
Heavy	117 (4.27%)	73 (3.55%)	44 (6.44%)	
Smoking status (*n* (%))				0.022
Never	1226 (44.73%)	944 (45.87%)	282 (41.29%)	
Past	726 (26.49%)	549 (26.68%)	177 (25.92%)	
Current	789 (28.79%)	565 (27.45%)	224 (32.80%)	
Follow-up duration (days)	1902.00 (826.00-3460.00)	1865.00 (778.50-3444.75)	2073.00 (1053.50-3474.00)	0.195
Incident T2D (*n* (%))				0.092
No	2518 (91.86%)	1901 (92.37%)	617 (90.34%)	
Yes	223 (8.14%)	157 (7.63%)	66 (9.66%)	

Notes: data are presented as *n* (%), mean ± SD, or median (IQR). Abbreviations: WC: waist circumference; BMI: body mass index; AST: aspartate aminotransferase; ALT: alanine aminotransferase; HDL-C: high-density lipoprotein cholesterol; GGT: gamma-glutamyl transferase; TG: triglyceride; TC: total cholesterol; FPG: fasting plasma glucose; HbA1c: hemoglobin A1c; DBP: diastolic blood pressure; SBP: systolic blood pressure; T2D: type 2 diabetes.

**Table 2:** Coefficients and lambda.1se value of the LASSO regression based on the training cohort.

Factors	Coefficients	Lambda.1se
Smoking status	0.065	0.018
WC (cm)	0.022	
FPG (mg/dL)	0.052	
HbA1c (mmol/mol)	0.199	

Abbreviations: LASSO: least absolute shrinkage and selection operator; WC: waist circumference; FPG: fasting plasma glucose; HbA1c: hemoglobin A1c.

**Table 3:** C-index in the nomogram based on training cohort and validation cohort.

	C-index (95% CI)	Dxy	aDxy	Variance	*Z* value	*P* value	*n*
Training cohort	0.839 (0.804, 0.874)	0.678	0.678	0.036	19.055	<0.001	2058
Validation cohort	0.822 (0.777, 0.868)	0.644	0.644	0.047	13.827	<0.001	683

## Data Availability

All datasets generated and/or analyzed during the present study are included in this published article and available in Dryad (http://www.datadryad.org/).
